# Hydrogen/oxygen therapy for the treatment of an acute exacerbation of chronic obstructive pulmonary disease: results of a multicenter, randomized, double-blind, parallel-group controlled trial

**DOI:** 10.1186/s12931-021-01740-w

**Published:** 2021-05-13

**Authors:** Ze-Guang Zheng, Wu-Zhuang Sun, Jie-Ying Hu, Zhi-Jun Jie, Jin-Fu Xu, Jie Cao, Yuan-Lin Song, Chang-Hui Wang, Jing Wang, Hui Zhao, Zhong-Liang Guo, Nan-Shan Zhong

**Affiliations:** 1 State Key Laboratory of Respiratory Disease, National Clinical Research Center for Respiratory Disease, Guangzhou Institute of Respiratory Health, Department of Respiratory and Critical Care Medicine, The First Affiliated Hospital of Guangzhou Medical University, 151 Yanjiang Road, Guangdong, China; 2 Department of Respiratory and Critical Care Medicine, The First Hospital of Hebei Medical University, Shijiazhuang, China; 3 Department of Respiratory and Critical Care Medicine, The Fifth People’s Hospital of Shanghai, Shanghai, China; 4 Department of Respiratory and Critical Care Medicine, Shanghai Pulmonary Hospital, Shanghai, China; 5 Department of Respiratory and Critical Care Medicine, Tianjin Medical University General Hospital, Tianjin, China; 6 Department of Respiratory and Critical Care Medicine, Zhongshan Hospital, Fudan University, Shanghai, China; 7 Department of Respiratory and Critical Care Medicine, Shanghai Tenth People’s Hospital, Shanghai, China; 8 Department of Respiratory and Critical Care Medicine, The First Affiliated Hospital of Zhengzhou University, Zhengzhou, China; 9 Department of Respiratory and Critical Care Medicine, Second Hospital of Shanxi Medical University, Taiyuan, China; 10 Department of Respiratory and Critical Care Medicine, Shanghai East Hospital of Tongji University, Shanghai, China

**Keywords:** Chronic obstructive pulmonary disease, Acute exacerbation, Hydrogen, Oxygen, Outcome

## Abstract

**Background:**

To investigate whether the administration of hydrogen/oxygen mixture was superior to oxygen in improving symptoms in patients with acute exacerbation of chronic obstructive pulmonary disease (AECOPD).

**Methods:**

This prospective, randomized, double-blind, controlled clinical trial in 10 centres enrolled patient with AECOPD and a Breathlessness, Cough, and Sputum Scale (BCSS) score of at least 6 points. Eligible patients were randomly assigned (in a 1:1 ratio) to receive either hydrogen/oxygen mixture or oxygen therapy. Primary endpoint was the change from baseline in BCSS score at day 7. Adverse events (AEs) were recorded to evaluate safety.

**Results:**

Change of BCSS score in Hydrogen/oxygen group was larger than that in Oxygen group (− 5.3 vs. − 2.4 point; difference: − 2.75 [95% CI − 3.27 to − 2.22], meeting criteria for superiority). Similar results were observed in other time points from day 2 through day 6. There was a significant reduction of Cough Assessment Test score in Hydrogen/oxygen group compared to control (− 11.00 vs. − 6.00, *p* < 0.001). Changes in pulmonary function, arterial blood gas and noninvasive oxygen saturation did not differ significantly between groups as well as other endpoints. AEs were reported in 34 (63.0%) patients in Hydrogen/oxygen group and 42 (77.8%) in Oxygen group. No death and equipment defects were reported during study period.

**Conclusions:**

The trial demonstrated that hydrogen/oxygen therapy is superior to oxygen therapy in patient with AECOPD with acceptable safety and tolerability profile. *Trial registration:* Name of the registry: U.S National Library of Medicine Clinical Trials; Trial registration number: NCT04000451; Date of registration: June 27, 2019-Retrospectively registered; URL of trial registry record: https://www.clinicaltrials.gov/ct2/show/study/NCT04000451?term=04000451&draw=2&rank=1.

**Supplementary Information:**

The online version contains supplementary material available at 10.1186/s12931-021-01740-w.

## Background

Chronic obstructive pulmonary disease (COPD) is a heterogeneous disease characterized by chronically poor airflow, which now has been a global disease with an estimated 63 million people worldwide [1]. Unfortunately, to date, there is no curative therapy for COPD, and these therapies are mostly palliative [2]. The disease progression of COPD is variable, with some patients having a relatively stable course, while others suffer relentless progression leading to severe breathlessness, frequent acute exacerbation of COPD (AECOPD) [3]. AECOPD are a frequent cause of admission to hospital and even intensive care unit, and mainly responsible for the mortality associated with the disease [4]. AECOPD were characterized by incompletely reversible [5], therefore, standard management mainly includes the bronchodilators, noninvasive ventilation (NIV) and oxygen therapy [6]. The ability of current medical treatments to reverse severe respiratory failure in these patients is limited [7]. Usually, oxygen therapy may induce the dangerous event in patients with COPD, such as hypercapnia [8]. Although NIV improves outcomes in patients with COPD and acute respiratory failure [9], persistent hypercapnia after an acute exacerbation is responsible for the early rehospitalization and excess mortality [10, 11]. Therefore, any alternative therapy likely to improve the oxygen therapy would be a valuable asset.

Therapeutic medical gas as pharmaceutical gaseous molecules are emerging as a novel and innovative therapeutic tool for COPD, including oxygen, nitrous oxide and helium [12, 13]. A prospective randomised controlled trial (RCT) has demonstrated that nitrous oxide reduces pulmonary hypertension in patients with COPD [14]. Besides, the inhalation of helium aid the reversal of airflow obstruction by reducing the resistance to flow in the airways and the work of breathing in severe COPD [15], since helium has a low density and molecular weight (MW). In recent years, molecular hydrogen has been accepted to have potential for preventive and therapeutic applications against many diseases due to its extensive effects, such as antioxidant, anti-inflammatory, anti-apoptotic and rapidly diffuses [16, 17]. Moreover, hydrogen is the lightest and smallest gas molecule, and more importantly, it found to function as an antioxidant to improve lung function [18]. Thus, we assumed that the inhalation of a hydrogen/oxygen mixture may be an alternative therapy. We attempted to combine oxygen and hydrogen therapy in patients with AECOPD. To allow mobility and easy use at home, a novel device named Hydrogen/Oxygen Generator with Nebulizer was used to provide the hydrogen/oxygen mixture. The efficacy of hydrogen/oxygen therapy has been demonstrated in patients with tracheal stenosis [19]. However, the clinical evidence for its efficacy and safety in upper airway obstruction might have been insufficient.

Here, we proposed a hypothesis that hydrogen/oxygen mixture may be superior to oxygen therapy in the improving respiratory symptoms for patient with AECOPD. Thus, the primary purpose of present study was to compare the efficacy of hydrogen/oxygen mixture produced by this novel Hydrogen/Oxygen Generator and oxygen alone in patients with AECOPD. The secondary objective was to assess its safety and tolerability.

## Methods

### Trial design

This was a prospective, multicentre, double-blind, randomized, controlled trial (registration number: NCT04000451) comparing the hydrogen/oxygen mixture therapy and oxygen alone therapy in patients with AECOPD. Patients were recruited from 10 centres in China. The trial was approved by the local ethics committee of all participating centres. All recruited patients provided written informed consent before participating in the trial. The study was conducted in accordance with Declaration of Helsinki.

### Subjects eligibility

Patients, aged 40 years or older, were eligible for this study if they had evidence of clinically acute exacerbation of COPD according to the diagnostic criteria [20, 21]. All patients has a baseline forced expiratory volume in 1 s (FEV_1_) less than 80% and FEV_1_/forced vital capacity (FVC) less than 70% in pulmonary function test. The AECOPD was defined as an increase in or new onset of at least two major COPD symptoms (wheezing, sputum production or sputum purulence), or one major COPD symptom plus at least one minor COPD symptom [fever, increase in respiratory rate and heart rate (≥ 20% from baseline), cough, wheezing rale and sore throat/rhinorrhea with 5 days] during at least 2 days consecutively and requiring any change of pharmacological treatment [21]. Patients were also required to have a baseline Breathlessness, Cough, and Sputum Scale (BCSS) score of at least 6 points.

Patient were ineligible if they have received intravenous or oral methylprednisolone (> 80 mg/day) or equivalent dose of other hormones during screen period or required continuous NIV during the index exacerbation. Besides, patients with other abnormalities of the thorax or the lung were also excluded. Additional exclusion criteria were malignant co-morbidities, severe cardiac diseases, diabetes, confirmed or suspected lung cancer, etc. Complete eligibility criteria are listed in the supplement (Additional file 1: Table S1).

### Intervention

**Fig. 1:**
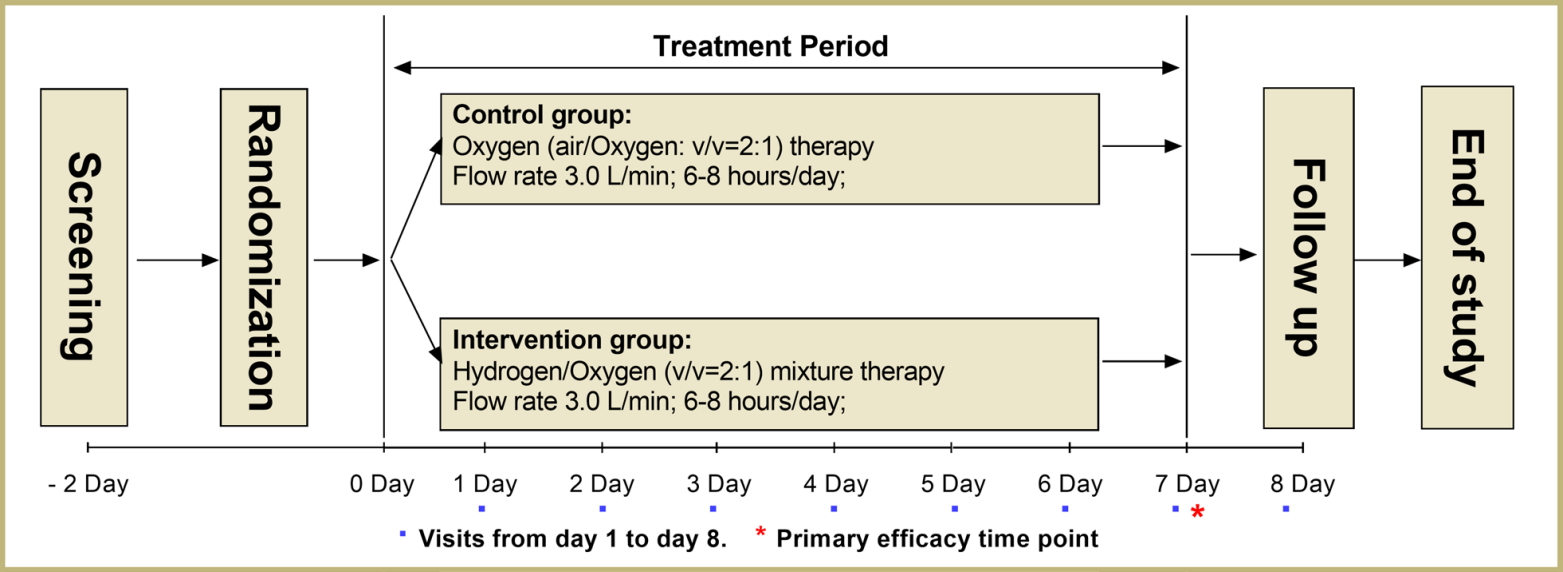
Study schema. Blue spots: follow-up time point; Red asterisk: Primary efficacy time point

All eligible patients were randomly assigned in a 1:1 ratio using a randomisation sequence created by a computer program, to receive either hydrogen/oxygen mixture therapy or oxygen alone therapy (Fig. 1). Treatment allocation was concealed from participants and study staff. Both hydrogen/oxygen mixture and oxygen were introduced via a nasal mask. Hydrogen/oxygen mixture therapy was delivered using a novel device, Hydrogen/Oxygen Generator with Nebulizer (AMS-H-01, Shanghai Asclepius Meditech Co., Ltd. China), at flow rate of 3.0 L/min and hydrogen/oxygen volume ratio of 2:1. Oxygen alone therapy was delivered using a Medical oxygen concentrator with molecular sieve (OLO-1, Shanghai Ouliang Medical Apparatus and Instruments Co., Ltd.) at flow rate of 3.0 L/min and air/oxygen volume ratio of 2:1. The oxygen-generator was re-designed to have consistent appearance with Hydrogen/Oxygen Generator with Nebulizer. The flow rate was set at 3.0 L/min for Hydrogen/Oxygen Generator (Hydrogen: 2.0 L/min; Oxygen: 1.0 L/min) and 3.0 L/min for Medical oxygen concentrator (air: 2.0 L/min; Oxygen: 1.0 L/min). During study, if oxygen saturation (SpO_2_) < 88% for patients in both two groups, additional oxygen supplementation was titrated to maintain SpO_2_ ≥ 88% by another oxygen concentrator. The oxygen titration was achieved by changes in the delivered gas flow rate. If SpO_2_ was still less than 88% when total oxygen flow rate reached 7.0 L/min, patients were allowed to withdraw from the study and administered with NIV or endotracheal intubation. Hydrogen/oxygen mixture or oxygen was administered for 7 consecutive days and from a minimum of 6 h/day to a maximum of 8 h/day.

**Fig. 2:**
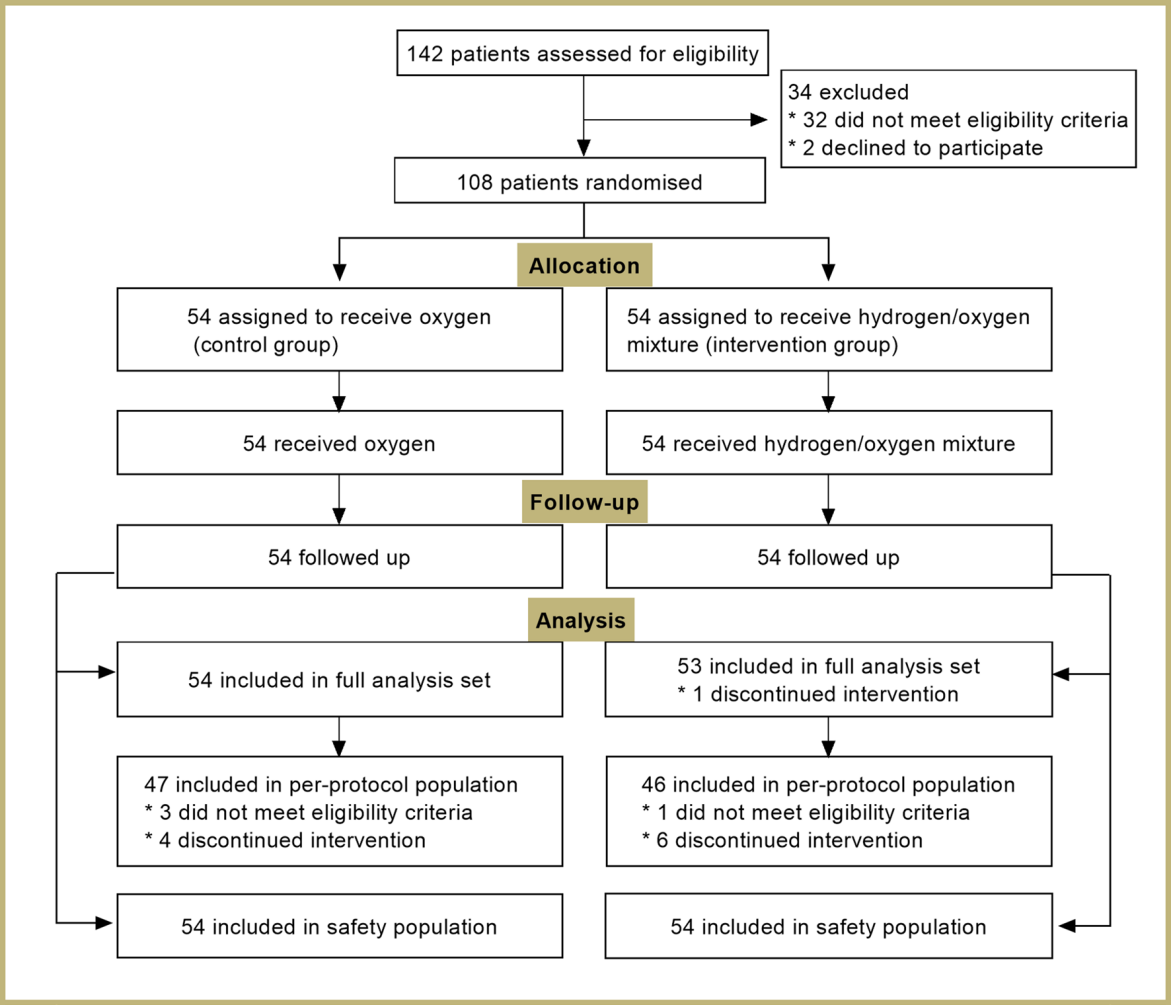
Patient flow diagram

### Outcomes and assessment

The primary efficacy endpoint was the change from baseline in BCSS score at day 7. The symptom severity of AECOPD was measured from baseline to day 7 via BCSS (0 = no symptoms, 12 = severely affected), with mean changes of 0.3 considered small, 0.6 moderate and > 1 substantial [22]. Secondary endpoints were the change in Cough Assessment Test (CAT) score as measured using CAT Questionnaire (categorical scale from 0 to 40; higher scores indicate more severely affected), and change in noninvasive oxygen saturation (SpO_2_). The exploratory endpoints were also investigated, including change in pulmonary function (FVC, FEV_1_ and FEV_1_/FVC), change in arterial blood gas while breathing room air [pH, arterial oxygen pressure (PaO_2_), arterial carbon dioxide pressure (PaCO_2_), bicarbonate [HCO_3_^−^)], additional oxygen inhalation, additional NIV, and instrument performance evaluation. The CAT score, pulmonary function, and arterial blood gas were measured at baseline and day 8. The SpO_2_, additional oxygen inhalation and NIV, instrument performance evaluation were recorded from baseline to each visit time point. Safety were assessed by the incidence of adverse events (AEs) or death, the changes of physical examinations, vital signs, laboratory data, and the incidence of equipment defects (refer the unreasonable risks that may endanger human health and life safety in normal use of medical devices during clinical trials). Any AEs were recorded during trial period. Severity of AEs was classified using the National Cancer Institute Common Terminology Criteria for Adverse Events version 4.03 [23].

### Statistical analysis

The sample size calculation for this superiority trial was based on the published data [22, 24] and our unpublished pilot data. We assumed that the predefined superiority margin was an absolute difference of 1.1 in the primary endpoint between groups. With a superiority limit of 0 on the relative scale, and assuming a loss to follow-up of 20%, 108 subjects (54 subjects per group) would be needed to provide 80% power with a 1-sided significance level of 2.5%.

All efficacy analyses were analyzed respectively in full analysis set (FAS) and per-protocol set (PPS). According to the intent-to-treat principle, FAS was defined as all randomized subjects who received any part of study treatment and received at least one evaluation of therapeutic effectiveness. PPS was defined as randomized subjects who completed the study and the absence of any major protocol violations. Safety assessment was analyzed in safety set (SS), which included all patients who had received at least 1 treatment.

Quantitative data were present as mean (standard deviation [SD]) or median (interquartile range [IQR]) as appropriate. Comparison of mean change in BCSS score and SpO_2_ at different time points between two groups were analyzed using repeated measures analysis of variance (ANOVA) with Bonferroni post hoc test. Analysis of CAT score, pulmonary function data and arterial blood gas data were performed using Students’ t-test or Wilcoxon rank sum test for between-group comparisons. Qualitative data, including additional oxygen inhalation, additional NIV, instrument performance evaluation and safety endpoints, were present as number (percentage) and analyzed by Fisher’s Exact Test or χ^2^ test as appropriate for between-group comparisons. The center effect was evaluated using PROC GLM of SAS ver. 9.2. Statistical significance was set at the two-sided significance level of 0.05 with 95% confidence intervals (CI). All statistical analyses were conducted using SAS software version 9.2 (SAS Institute Inc, USA).

## Results

### Patient characteristics

Of the 142 subjects screened, 108 were eligible and randomly assigned to treatment (Fig. 2). There were 54 patients randomized to hydrogen/oxygen mixture therapy and 54 patients to oxygen therapy alone. Of 108 eligible patients enrolled in this study, a total of 107 patients were included in FAS analysis (Oxygen group: n = 54; Hydrogen/oxygen group: n = 53). One patient was excluded from FAS population due to discontinued intervention (consent withdraw). Furthermore, 93 patients completed the study and were included in PPS analysis (Oxygen group: n = 47; Hydrogen/oxygen group: n = 46). The reasons for not including in PPS analysis were the delayed recognition that patient did not meet the eligibility criteria (n = 4) and discontinued intervention (n = 10). Two patients in each group withdraw due to AEs. Baseline characteristics were listed in Table 1. The two groups were well balanced for the baseline variables, including age, gender, disease history and clinical symptoms. Included patients in each centre were listed in Additional file 1: Table S2.

**Table 1:** Baseline characteristics of patients in the full analysis set population

Characteristic	Oxygen group (n = 54)	Hydrogen/oxygen group (n = 53)	P value
Age-year, mean(SD)	69.8 (8.23)	69.7 (7.72)	0.959
Male sex-n (%)	46 (85.2%)	48 (90.6%)	0.556
Height-cm, mean (SD)	164.7 (6.86)	167.0 (6.98)	0.092
Weight-kg, mean (SD)	60.05 (9.86)	63.52 (12.87)	0.120
BMI-kg/m^2^,mean (SD)	22.15 (3.47)	22.74 (4.09)	0.421
History of Smoking, yes-n (%)	42 (77.8%)	45 (84.9%)	0.667
Average smoking amount/day, mean (SD)	16.4 (8.97)	24.8 (16.40)	0.127
Disease history, yes-n (%)	51 (94.4%)	49 (92.5%)	0.716
Clinical symptoms, yes-n (%)			
Aggravated wheezing	52 (96.3%)	48 (90.6%)	0.270
Increased sputum production	37 (68.5%)	35 (66.0%)	0.838
Sputum purulence	24 (44.4%)	21 (39.6%)	0.696
Fever	2 (6.9%)	1 (3.8%)	> 0.999
Aggravated cough	28 (96.6%)	24 (92.3%)	0.598
Increased wheezing rale	7 (24.1%)	6 (23.1%)	> 0.999
Increased breathing and heart rate (≥ 20% from baseline)	0 (0.0%)	1 (3.8%)	0.473
Sore throat or rhinorrhea with 5 days	1 (3.4%)	0 (0.0%)	> 0.999

*BMI* body mass index, *SD* standard deviation

The mean time of instrument exposure was 6.4 days (range 1–7 days) for the Hydrogen/oxygen group and 6.4 days (range 1–7 days) for the Oxygen group, with no significant between-group differences. The mean treatment period ranged from 364.9 to 375.4 min/day in Hydrogen/oxygen group and 366.0–378.5 min/day in Oxygen group, without significant between-group differences. The compliance between two groups were generally balanced.

### Primary endpoint

**Fig. 3:**
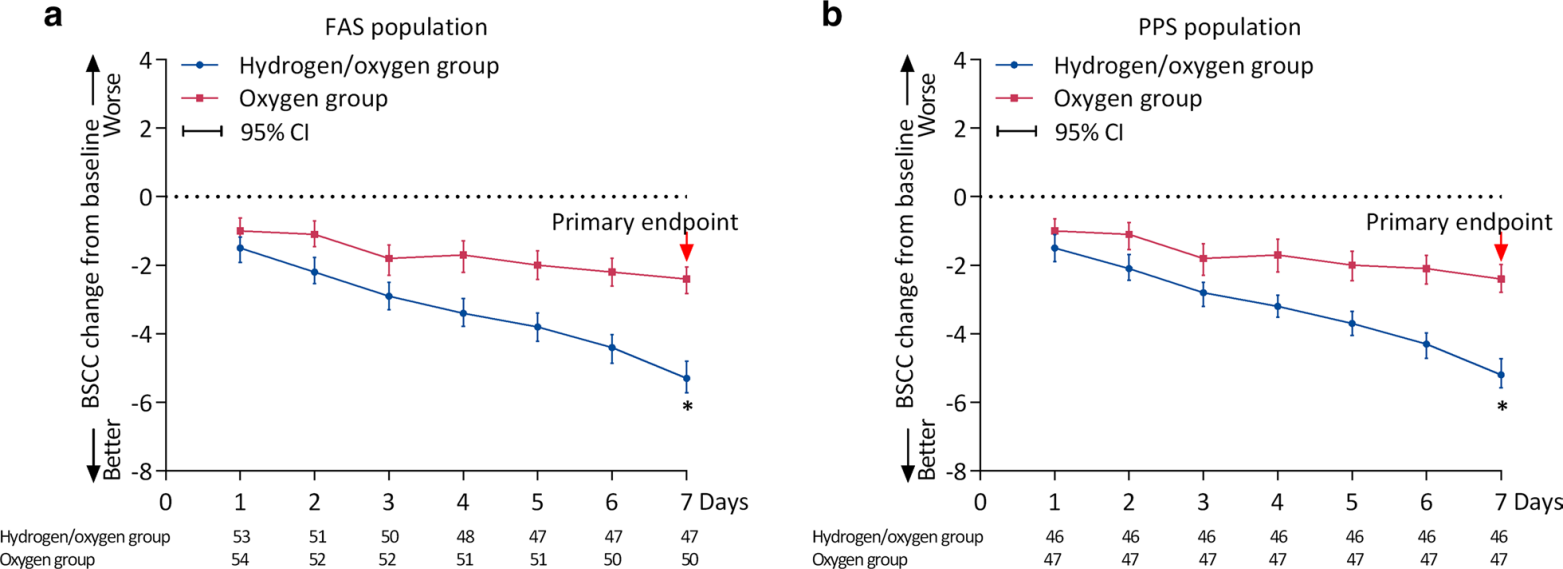
Seven-days changes from baseline in BCSS score in FAS (**a**) and PPS (**b**) population. *BCSS* breathlessness, Cough and Sputum Scale, *FAS* full analysis set, *PPS* per-protocol set. *p < 0.05. Red asterisk represent that the BCSS score change from baseline at day 7 is the primary efficacy endpoint

For the primary endpoint in FAS population, the change from baseline in BCSS score was − 5.3 (range − 10 to − 1) in the Hydrogen/oxygen group and − 2.4 (range − 6 to 0) in Oxygen group (Fig. 3a). The difference in the primary endpoint was − 2.75 (95% CI − 3.27 to − 2.22), with the upper confidence limit not more than the superiority limit of 0. Similarly, in the PPS population, the change from baseline in BCSS score was − 5.2 (range − 8 to − 1) in the Hydrogen/oxygen group and − 2.69 (range − 6–0) in Oxygen group (Fig. 3b). The difference in the primary endpoint was − 2.69 (95% CI − 3.21 to − 2.17), with the upper confidence limit not more than the superiority limit of 0. After treatment, for patients either receiving hydrogen/oxygen or air/oxygen, the reduction of BCSS score from baseline reached significance form day 1, and showed a continuous trend of reduction (Fig. 3). The repeated-measures ANOVA with Bonferroni post hoc test showed that patients who received hydrogen/oxygen had a more significant improvement in BCSS score over time (from day 1 to day 7) compared to patients received oxygen (*p* < 0.0001), and the time by group interaction effect was also significant (*p* < 0.0001). But, no plateau period was observed in the tendency of symptom improvement during the entire study (Fig. 3a, b). Notably, improvement from baseline in BCSS score reached significance in patients receiving hydrogen/oxygen therapy compared with controls from day 2 to day 7 (Fig. 3a, b). No center effect was identified using PROC GLM model (*p* = 0.14).

### Secondary endpoints

**Fig. 4:**
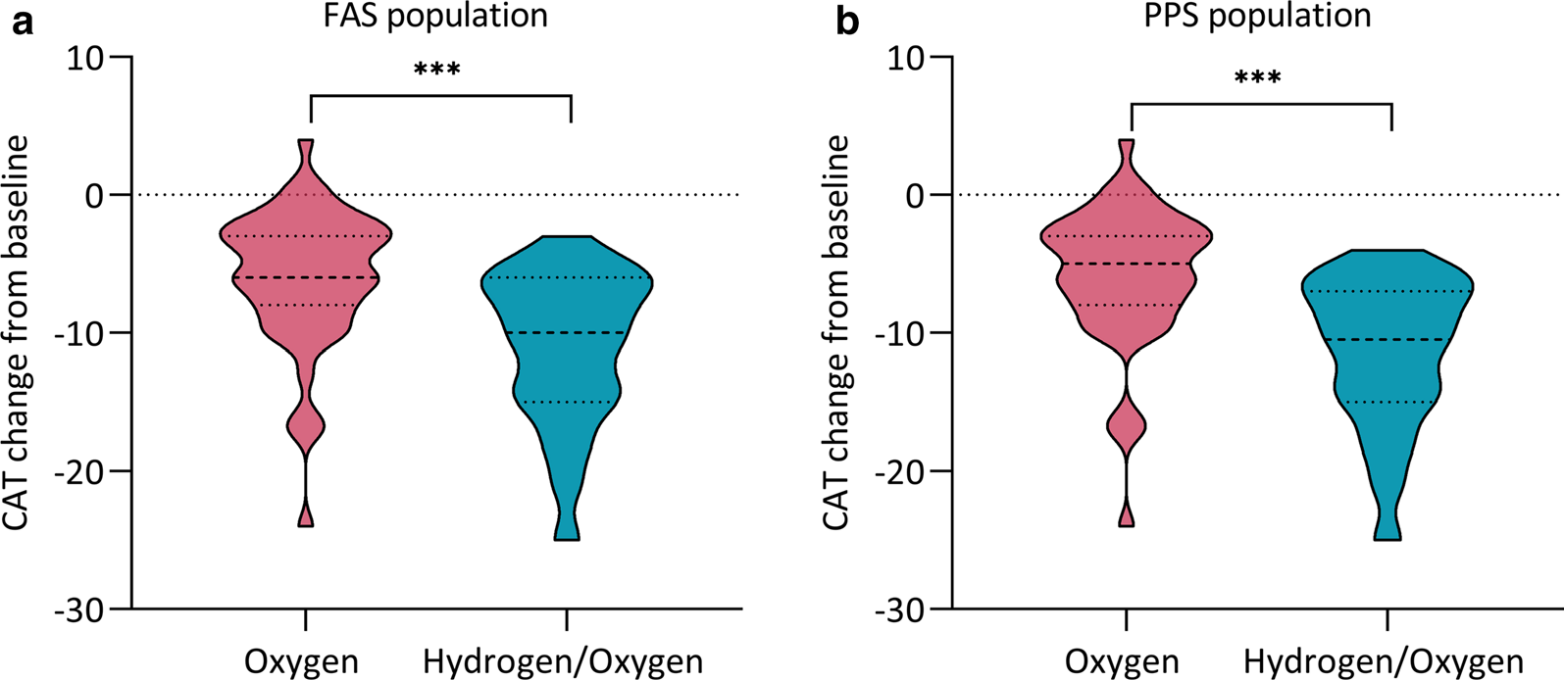
Changes from baseline in CAT score in in FAS (**a**) and PPS (**b**) population. *CAT* Cough Assessment Test, *FAS* full analysis set, *PPS* per-protocol set. ***p < 0.001

With regard to CAT score, there was a statistically significant reduction in the Hydrogen/oxygen group [− 11.00 (95% CI, − 12.60 to − 9.48)] compared to the control [− 6.00 (95% CI, − 7.46 to − 4.61)] in FAS population (*p* < 0.001, Fig. 4a). The data in PPS population were also consistent with these results [− 11.40 (95% CI − 12.99 to − 9.79) vs. − 5.90 (95% CI − 7.37 to − 4.38), *p* < 0.001, Fig. 4b].

**Fig. 5:**
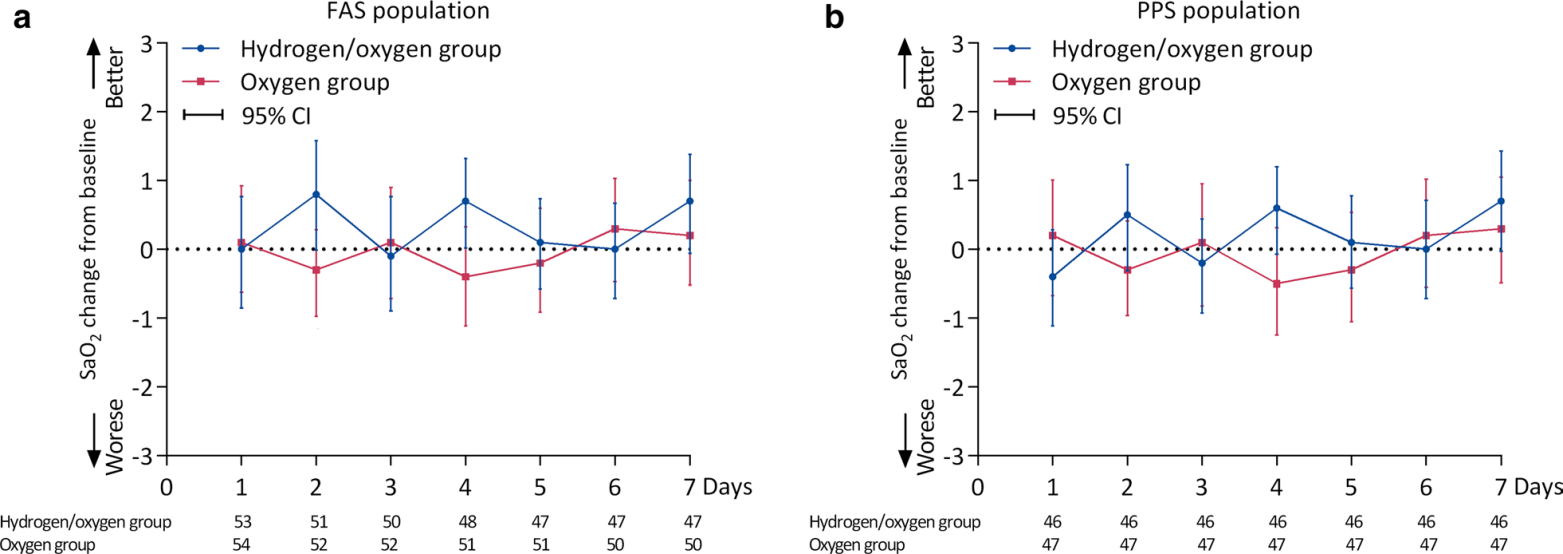
Seven-days changes from baseline in SpO_2_ in FAS (**a**) and PPS (**b**) population. SpO_2_, noninvasive oxygen saturation; *FAS* full analysis set, *PPS* per-protocol set. *p < 0.05

The changes from baseline in SpO_2_ were shown in Fig. 5. There was no effect of time (*p* = 0.169) or group (*p* = 0.805) on changes from baseline in SpO_2_. However, there were time by group interactions with respect to the changes from baseline in SpO_2_ (*p* < 0.0001). Similar results were found in PPS population.

### Exploratory endpoints

In FAS population, changes from baseline in the pulmonary function parameters did not differ significantly between treatment groups (Table 2), including FVC (*p* = 0.309), FEV_1_ (*p* = 0.769) and FEV_1_/FVC (*p* = 0.536). In addition, the arterial blood gas was measured at day 8 after initial treatment to evaluate the arterial oxygenation. The patients in both treatment groups did not differ in terms of the arterial oxygenation parameters (Table 2), with no significant differences in pH (*p* = 0.700), PaO_2_ (*p* = 0.461), PaCO_2_ (*p* = 0.160), and HCO_3_^−^ (*p* = 0.136). The consistent results of pulmonary function and arterial oxygenation parameters were observed in the PPS population (Table 2). No patient received any other oxygen inhalation or noninvasive ventilation during study period, without significant between-group differences. Two systems used for therapy were evaluated favorably by all patients.

**Table 2:** Exploratory endpoints (change from baseline after treatment) in FAS and PPS population

Exploratory endpoints	Mean change from baseline (95% CI) in FAS population			Mean change from baseline (95% CI) in PPS population		
	Oxygen group	Hydrogen/oxygen group	P value	Oxygen group	Hydrogen/oxygen group	P value
Pulmonary function						
FVC	0.18 (0.07, 0.29)	0.10 (− 0.01,0.21)	0.309	0.19 (0.08, 0.31)	0.10 (− 0.01, 0.21)	0.250
FEV_1_	0.12 (0.06, 0.19)	0.11 (0.04, 0.18)	0.769	0.13 (0.06, 0.19)	0.11 (0.03, 0.18)	0.698
FEV_1_/FVC (%)	1.52 (− 0.40, 3.44)	2.45 (− 0.10, 4.99)	0.563	1.22 (− 0.83, 3.29)	2.29 (− 0.39, 4.97)	0.529
Arterial blood gas						
pH	0.001 (− 0.013, 0.014)	0.004 (− 0.009, 0.018)	0.700	0.001 (− 0.013, 0.016)	0.003 (− 0.010, 0.017)	0.875
PaO_2_ (mmHg)	− 0.45 (− 5.65, 4.75)	3.46 (− 5.77, 12.70)	0.461	1.39 (− 3.87, 6.65)	4.46 (− 5.74, 14.66)	0.592
PaCO_2_ (mmHg)	0.71 (− 1.09, 2.51)	− 1.09 (− 2.88, 0.71)	0.160	0.53 (− 1.42, 2.48)	-0.55 (− 2.44, 1.34)	0.424
HCO_3_^−^ (mmol/L)	0.61 (− 0.39, 1.62)	− 0.36 (− 1.20, 0.48)	0.136	0.56 (− 0.49, 1.61)	− 0.14 (− 1.03, 0.76)	0.315

*FVC* forced vital capacity, *FEV*_*1*_ forced expiratory volume in 1 s, *PaO*_*2*_ arterial oxygen pressure, *PaCO*_*2*_ arterial carbon dioxide pressure, *HCO*_*3*_^*−*^ bicarbonate, *95% CI* 95% confidence interval, *FAS* full analysis set, *PPS* per-protocol set

### Safety analysis

Summary of adverse events was shown in Table 3. Overall, AEs were reported in 34 (63.0%) patients in Hydrogen/oxygen group and 42 (77.8%) patients in Oxygen group, without statistic difference (*p* = 0.140). The common AEs in two groups included upper respiratory infection (Hydrogen/oxygen: 5.6% vs. Oxygen: 5.6%) and hypokalemia (3.7% vs. 5.6%). The majority of AEs in two groups were mild or moderate in severity (Grade 1 or 2), and only a small percentage of patients experienced severe AEs (Hydrogen/oxygen: 3.7% vs. Oxygen: 13.0%, *p* = 0.161). In the Hydrogen/oxygen group, device-related AEs occurred in 2 patients (3 events), including dizziness (mild), nasal mucosa injury (mild), and aggravated wheezing (moderate). In the Oxygen group, device-related AEs occurred in 3 patients (3 events), including agrypnia (moderate), abdominal distention (mild) and aggravated wheezing (moderate). In addition, two groups respectively reported 2 cases of AEs leading to withdrawal (Hydrogen/oxygen: 1 polypnea and 1 heart failure; Oxygen: 1 agrypnia and 1 cheat discomfort), without statistic difference. All AEs were resolved with treatment interruption and symptomatic treatment. No notable changes were observed in physical examinations, vital signs, liver and kidney functions. No death and equipment defects were reported during the study period.

**Table 3:** Summary of adverse events in safety population

Group	Oxygen group (n = 54)	Hydrogen/oxygen group (n = 54)
Number of patients, n (%)		
Any adverse events	42 (77.8%)	34 (63.0%)
Main AEs		
Hypokalemia	3 (5.6%)	2 (3.7%)
Abnormal liver function	3 (5.6%)	1 (1.9%)
Upper respiratory infection	3 (5.6%)	3 (5.6%)
Elevated C-reactive protein	2 (3.7%)	4 (7.4%)
Elevated blood pressure	2 (3.7%)	2 (3.7%)
Cough	2 (3.7%)	2 (3.7%)
Phlegm-retention	3 (5.6%)	0 (0.0%)
Wheezing	4 (7.4%)	0 (0.0%)
Agrypnia	2 (3.7%)	2 (3.7%)
Any severe adverse events	7 (13.0%)	2 (3.7%)
Abnormal liver function	1 (1.9%)	0 (0.0%)
Infection (lung, upper respiratory)	3 (5.6%)	0 (0.0%)
Bacterial infection	1 (1.9%)	0 (0.0%)
Pulmonary inflammation	0 (0.0%)	1 (1.9%)
Wheezing	2 (3.7%)	0 (0.0%)
Ileus	0 (0.0%)	1 (1.9%)
Any device-related adverse events	3 (5.6%)	2 (3.7%)
Any adverse events leading to withdrawal	2 (3.7%)	2 (3.7%)
Number of events, n		
Any adverse events	82	63
An severe adverse event	7	2
Any device-related adverse events	3	3
Upper respiratory tract infection	31(14.69)	26(20.31)

## Discussion

To our knowledge, this is the first multicenter, randomized, controlled trial investigating the efficacy of hydrogen/oxygen mixture in patients with AECOPD. Our direct comparison with the oxygen therapy which used widely in AECOPD has established the better efficacy of hydrogen/oxygen therapy in the improvement of respiratory function for the treatment of AECOPD. Meanwhile, the safety analysis demonstrated that hydrogen/oxygen therapy has acceptable tolerability.

Long-term home oxygen therapy has been proved to improve survival in patients with COPD [25, 26]. However, oxygen therapy in exacerbations of COPD can be both helpful and harmful [27]. Previously, Liu et al. have proposed a hypothesis that hydrogen may be a unique, effective, and specific treatment for COPD, due to its special advantages [28]. Herein, a novel Hydrogen/Oxygen Generator was developed to investigate the effect of hydrogen/oxygen mixture on AECOPD. The most striking finding of this study is that the inhalation of hydrogen/oxygen mixture is superior to oxygen alone in patients with AECOPD. The management of the key symptoms of COPD, such as breathlessness, cough and sputum, is the important treatment objective [29]. We therefore used the patient-reported assessment instrument BCSS to determine the therapeutic effect on patient symptoms. As a consequence, improvements from baseline in BCSS score were better in patients receiving hydrogen/oxygen mixture compared with oxygen alone. Furthermore, during the entire study period, hydrogen/oxygen therapy resulted in a more significant sustained reduction in BCSS score than oxygen therapy. Meanwhile, according to the tendency for symptoms to gradually improve, we speculated that the therapeutic effect of hydrogen/oxygen mixture might be associated with the total time and dose of hydrogen inhalation, suggesting that an optimized design with gradient of inhalation time and dose was needed. More exhilaratingly, no plateau period was observed in the tendency of symptom improvement during the entire study, especially in patients receiving hydrogen/oxygen therapy. In addition, after adjusting for main residual confounding variables on the main outcome, multivariate analysis further verified that intervention was the independent risk factor of substantial improvement in patients with AECOPD (OR = 0.033, 95% CI 0.009–0.117, *p* < 0.001, Additional file 2: Fig. S1), indicating that hydrogen/oxygen therapy were protective for the improvement of AECOPD. Overall, this phenomenon implicated that hydrogen/oxygen therapy may have greater potential to improve symptoms of AECOPD. Thereby, further investigations with the optimized treatment course as well as long-term follow-up are imperative.

In addition, CAT as a cough-specific quality-of-life questionnaire was used to assessing and monitoring cough in patients [30]. The better improvement in CAT score after the inhalation of hydrogen/oxygen mixture supported our primary analysis, further indicating the superiority of hydrogen/oxygen therapy. Similarly, the results of previous study in tracheal stenosis demonstrated that the inhalation of hydrogen/oxygen mixture reduce the inspiratory effort, which further supports our present study [19]. However, with regard to the exploratory endpoints, both pulmonary function, oxygenation and acid–base balance were comparable in two groups during treatment period. In fact, numerous evidences have revealed the therapeutic effects of hydrogen in a variety of animal disease models and human patients, and meanwhile indicated that hydrogen are comparable to the traditional therapeutic gases regimens including oxygen and hydrogen sulfide [31], which was consisted with our present study. Thus, we could conclude that hydrogen/oxygen therapy has the potential to be a novel and effective treatment for COPD.

The most striking finding of present study was the superiority of hydrogen/oxygen therapy in patient with AECOPD, accompanied by acceptable safety and tolerability profile. The molecular properties of hydrogen might explain why hydrogen/oxygen mixture is superior to oxygen alone in the therapy of AECOPD. We speculated that its superiority in the improvements of key symptoms of AECOPD may be attributable to its physical properties, anti-inflammatory, antioxidant and rapid cellular diffusion features [28]. Primarily, hydrogen has similar physical characteristics with gaseous helium, therefore, the inhalation of hydrogen would exert similar effects on the reduction of airway resistance. Previously, it is reported that the hydrogen/oxygen could reduce the inspiratory effort quickly in 100 s [19]. Accordingly, we assumed that the early improvements of symptoms were mainly due to the physical properties of hydrogen, in which its low density could reduce the resistance to flow in the airways, and in turn decrease the work of breathing. Subsequently, its biological functions including anti-inflammatory, antioxidant act in several hours, further promoting the improvement of symptoms. Mechanically, inflammation and oxidative stress (OS) participated in the pathogenesis of exacerbations of COPD [32–34]. The imbalance between oxidative stress and antioxidative capacity is thought to play an important role in the development and progression of COPD [35]. Thus, suppression of the inflammatory response, oxidative stress and antioxidant therapies is a logical approach to the treatment of COPD [36–38]. Patients with COPD exhibit increased oxidant production, such as hydrogen peroxide (H_2_O_2_) in the airways and that oxidant production increases further during exacerbations [39]. H_2_O_2_ could convert to very reactive ·OH in the presence of catalytically active metals [40], while ·OH is the major cause of the oxidation and biomolecules destruction by the direct reaction or by triggering the chain reaction of free radicals [41]. Previous study demonstrated that hydrogen did not change the cellular levels of O_2_·^−^ and H_2_O_2_, but significantly decreased levels of ·OH, this means that hydrogen is mild enough neither to disturb metabolic redox reactions nor to affect ROS that function in cellular signaling [16]. Hydrogen has previously been shown to reduce inflammatory factors, oxidative stress and reactive oxygen species in the patients with rheumatoid arthritis and related diseases [42, 43]. Animal studies also provided evidences for the anti-inflammatory and antioxidant action of hydrogen in various diseases, such as the pulmonary hypertension and COPD [44, 45]. Collectively, its favourable anti-inflammatory and antioxidant in the airway could explain the superior effects on the course of symptom during COPD exacerbation. Furthermore, hydrogen is a small molecule with rapid cellular diffusion features that can easily dissipate throughout the body and cells [46], which may contribute to the fast-acting of these effects. Nevertheless, this explanation cannot be confirmed from present study because we did not measure levels of these inflammatory and oxidative factors during treatment period. Besides, except that hydrogen may be a replacement of helium in the reduction of airway resistance, the exciting thing is that the hydrogen might be able to solve the inconvenience and high cost of helium in practice.

The treatment-related AEs are clearly an area of main concern when considering the development of a new therapeutic regimen. In this trial, we found that both hydrogen/oxygen and oxygen therapy regimens present acceptable safety and tolerability profile. Although approximately more than 60% patients experienced AEs in two groups, the majority of AEs were mild or moderate in severity. The most common AEs from hydrogen/oxygen therapy were upper respiratory infection and hypokalemia, which were also resulted from oxygen alone therapy. Similarly, most of these AEs were known and have also been reported in study on helium/oxygen therapy [15]. In fact, as reported in other publications, these AEs such as hypokalemia, nausea, increased intestinal gas, etc. were occurred frequently in the COPD treatment [47, 48], which were not exclusive to hydrogen/oxygen therapy. There results indicated that the hydrogen would not increase the risk of AEs in the AECOPD treatment. Notably, all AEs were controllable, tolerable and all resolved soon with treatment interruption and symptomatic treatment. Additionally, no notable changes in laboratory test and physical examinations suggested that administration of hydrogen did not interfere with the vital signs and somatic function, which was consistent with the results of previous studies [19, 49]. More importantly, we observed that device-related AEs were infrequent and only a few occurrences in each group, indicating that both the two devices were safe enough. Overall, from the safety perspective, both therapeutic regimens have the acceptable safety, implicating that hydrogen/oxygen produced by Hydrogen/Oxygen Generator can be inhaled safely in patients with AECOPD. We boldly speculate that hydrogen/oxygen therapy may be an alternative to long-term oxygen therapy at home.

Although this study demonstrated important findings, it also has several limitations. First, the lack of a control design with helium/oxygen for this trial is a potential criticism. In fact, the use of oxygen as control was considered because the helium-producing devices have not been used in AECOPD therapy in China. Meanwhile, the voice alteration caused by helium inhalation would break the double-blind design. Thus, given that the definite effect of oxygen in COPD [50, 51], a medical oxygen concentrator was selected as the positive control by unifying the device appearance. Second, the explosive risk of this device was a primary concern for this this trial. However, this problem has been adequately tested. Through testing in a confined space, the maximum hydrogen concentration was 0.8% after 2 h of continuous operation, which was far below the explosion limits of hydrogen in the air (4%) [19]. Also, the relatively short study period was a limitation of this trial, which may not evaluate the long-term benefit of hydrogen/oxygen therapy. In addition, the study had a relative low sample size. But even so, the study has sufficient power reach the intended target for the primary endpoint. Thus, the large-scale trials are in progress or planned on the basis of the findings in this study.

## Conclusion

In the first trial to date on hydrogen/oxygen therapy for AECOPD treatment, compared with oxygen, the inhalation of hydrogen/oxygen mixture resulted in a more significant improvement of AECOPD symptoms, including breathlessness, cough and sputum, with acceptable safety and tolerability profile. The findings suggested that the hydrogen/oxygen therapy was feasible and well tolerated for patients with AECOPD, having potential to be used as an alternative emergency management for AECOPD.

## Supplementary Information

**Additional file 1:** **Table S1.** Complete eligibility criteria. **Table S2.** The included patients in each centre.

**Additional file 2: Figure S1.** Forest plot of the multivariate analysis. Change from baseline in BCSS score >1 was identified as substantial improvement and ≤1 as non-substantial improvement, which were used as outcomes in logistics regression analysis.

## Data Availability

The datasets generated and/or analysed during the current study are not publicly available, but are available from the corresponding author on reasonable request.
