# Supplementary material for: Hydrogen/oxygen therapy for the treatment of an acute exacerbation of chronic obstructive pulmonary disease: results of a multicenter, randomized, double-blind, parallel-group controlled trial
Source: Respir Res. 2021 May 13;22:149. doi: 10.1186/s12931-021-01740-w (PMC8120708; doi:10.1186/s12931-021-01740-w)
Supplement: Supplementary file 1 — Additional file 1: Table S1. Complete eligibility criteria. Table S2. The included patients in each centre. [file 12931_2021_1740_MOESM1_ESM.docx]

**Additional Table S1. Complete eligibility criteria.**

| **Inclusion criteria** |
| --- |
| All subjects participating in this clinical trial must meet all of the following criteria:  1) Meets the diagnostic criteria for COPD [1]: The forced expiratory volume in 1 second is <70% of the forced vital capacity (FEV1/FVC%), FEV1 <80% after the use of a bronchodilator in the pulmonary function test;  2) Meets the diagnostic criteria for AECOPD [2]: Patient's conditions continue to worsen compared with the stable period, exceeding normal daily changes. COPD patients have an acute onset and conventional treatment needs to be adjusted. Continued worsening of at least 2 of the following 3 clinical symptoms: ① Aggravated wheezing; ② Increased sputum volume; ③ Sputum purulence; Or at least 1 of the above 3 symptoms is observed plus any 1 of the following 5 symptoms: ① Fever; ② A 20% increase in respiratory rate and heart rate from baseline; ③ Aggravated coughing; ④ Sore throat and rhinorrhea in past 5 days; ⑤ Increased wheezing rale;  3) >40 years old male or female with normal autonomous judgment;  4) AECOPD patient requiring hospitalization for treatment;  5) BCSS score ≥6 at time of enrollment;  6) Voluntary participation in the trial and have signed the informed consent. |
| **Exclusion criteria** |
| 1) Received >80mg/d intravenous or oral methyprednisolone or equivalent dose of other hormones or require continuous noninvasive ventilation due to severe conditions;  2) Has significant disease other than COPD, that is, a disease that, according to the investigator's judgment, can cause a subject to be at risk due to participation in the study, or affect the results of the study and the ability of the subject to participate in the study;  3) Other active respiratory diseases, such as active tuberculosis, lung cancer, bronchiectasis (CT shows repeated acute exacerbation caused by bronchiectasis), sarcoidosis, idiopathic interstitial pulmonary fibrosis (IPF), primary pulmonary arterial hypertension, and uncontrolled sleep apnea (the severity of the disease will affect the implementation of the study according to the investigator's evaluation);  4) Pulmonary rehabilitation: subjects who will participate in a pulmonary rehabilitation program during this study;  5) History of severe heart diseases such as acute myocardial infarction, congestive failure (NYHA grade III or higher), severe arrhythmia, and other acute heart disease;  6) Severe primary diseases of important organs or systems such as acute stroke, moderate or higher hypertension after treatment, active gastric ulcer, diabetes (severe complications), and malignancy;  7) Patients with confirmed or suspected lung cancer;  8) History of resection of one or more lung lobes;  9) Limited understanding and poor compliance;  10) Lack of legal capacity or has restricted legal capacity;  11) Participation in clinical trial of other drug or medical device and has not reached endpoint within 30 days prior to screening;  12) Pregnant or breastfeeding women and women with childbearing potential who are unwilling to take effective contraceptive measures during the study;  13) Mental or physical disabilities;  14) Suspected or confirmed history of alcohol or drug abuse;  15) Patients with known intolerance to inhalation therapy;  16) AST and ALT levels are 3 times higher than the upper limit of normal level, and creatinine is ≥176.8mmol/L;  17) Shock or other hemodynamic instability;  18) Infectious diseases (hepatitis A, hepatitis B, AIDS, and tuberculosis) and active connective tissue diseases;  19) >5 days of intravenous steroid therapy after an acute attack;  20) Use of non-expectorants and antioxidants, including large doses of vitamin C and vitamin E;  21) Subjects who are not suitable for participation in this study in the opinion of investigator. |

Additional Table S2. The included patients in each centre.

| Centre | FAS (n=107) | | PPS (n=93) | | SS (n=108) | |
| --- | --- | --- | --- | --- | --- | --- |
|  | Hydrogen/oxygen group (n=53) | Oxygen group (n=54) | Hydrogen/oxygen group (n=46) | Oxygen group (n=47) | Hydrogen/oxygen group (n=54) | Oxygen group (n=54) |
| 1 | 10 | 10 | 9 | 9 | 10 | 10 |
| 2 | 6 | 6 | 6 | 6 | 6 | 6 |
| 3 | 2 | 2 | 1 | 2 | 2 | 2 |
| 4 | 12 | 12 | 10 | 12 | 12 | 12 |
| 5 | 4 | 4 | 4 | 4 | 4 | 4 |
| 6 | 4 | 4 | 2 | 3 | 4 | 4 |
| 7 | 3 | 4 | 2 | 1 | 4 | 4 |
| 8 | 4 | 4 | 4 | 2 | 4 | 4 |
| 9 | 2 | 2 | 2 | 2 | 2 | 2 |
| 10 | 6 | 6 | 6 | 6 | 6 | 6 |
